# YOLO-AMM: A Real-Time Classroom Behavior Detection Algorithm Based on Multi-Dimensional Feature Optimization

**DOI:** 10.3390/s25041142

**Published:** 2025-02-13

**Authors:** Yi Cao, Qian Cao, Chengshan Qian, Deji Chen

**Affiliations:** 1 School of Internet of Things Engineering, Wuxi University, Wuxi 214105, China; caoyi@cwxu.edu.cn (Y.C.); dejichen@cwxu.edu.cn (D.C.); 2 School of Automation, Nanjing University of Information Science & Technology, Nanjing 210044, China; c1519958281@163.com; 3 Jiangsu Foreign Expert Lab, Wuxi 214105, China

**Keywords:** YOLOv8, classroom behavior detection, AEFF, MFFN

## Abstract

Classroom behavior detection is a key task in constructing intelligent educational environments. However, the existing models are still deficient in detail feature capture capability, multi-layer feature correlation, and multi-scale target adaptability, making it challenging to realize high-precision real-time detection in complex scenes. This paper proposes an improved classroom behavior detection algorithm, YOLO-AMM, to solve these problems. Firstly, we constructed the Adaptive Efficient Feature Fusion (AEFF) module to enhance the fusion of semantic information between different features and improve the model’s ability to capture detailed features. Then, we designed a Multi-dimensional Feature Flow Network (MFFN), which fuses multi-dimensional features and enhances the correlation information between features through the multi-scale feature aggregation module and contextual information diffusion mechanism. Finally, we proposed a Multi-Scale Perception and Fusion Detection Head (MSPF-Head), which significantly improves the adaptability of the head to different scale targets by introducing multi-scale feature perception, feature interaction, and fusion mechanisms. The experimental results showed that compared with the YOLOv8n model, YOLO-AMM improved the mAP0.5 and mAP0.5-0.95 by 3.1% and 4.0%, significantly improving the detection accuracy. Meanwhile, YOLO-AMM increased the detection speed (FPS) by 12.9 frames per second to 169.1 frames per second, which meets the requirement for real-time detection of classroom behavior.

## 1. Introduction

In recent years, with the rapid development of artificial intelligence technology, target detection technology has made significant progress in computer vision, especially in pedestrian detection [1], traffic monitoring [2], and driverless applications [3]. However, effectively using this technology in the field of education to assess students’ classroom behavior and improve the quality of teaching is still a challenge that needs to be solved. As an efficient data acquisition and processing tool, intelligent visual sensors show great potential in classroom behavior monitoring. By collecting classroom video data in real time and combining it with improved intelligent algorithms for behavioral analysis, these sensors can provide efficient and accurate monitoring to help teachers promptly adjust their teaching strategies and improve classroom interactivity and teaching effectiveness. Therefore, improving the accuracy and real-time performance of classroom behavior monitoring systems has become a key issue in education.

The academic contributions of this study are mainly reflected in the following two aspects: first, a fine-grained behavior detection algorithm for educational scenarios is proposed, which provides a new technical path for solving the target detection problem in complex teaching environments; second, the detection performance of the algorithm is significantly improved by the innovative design of adaptive feature fusion, multi-dimensional feature flow network, and multi-scale perceptual fusion detection head. Compared with the existing methods, the algorithm proposed in this study has stronger adaptability and better real-time performance in complex classroom environments, providing solid technical support for the development and broad application of intelligent visual sensors. This research solves the bottleneck of the current classroom behavior detection technology in terms of accuracy and efficiency. It promotes the technological advancement of intelligent educational systems, which has important application value and broad prospects.

Classroom behavior recognition methods focus on deep learning models, particularly Convolutional Neural Networks (CNNs) and target detection algorithms such as the YOLO series. Zhou et al. [4] employed the CNN-10 model to detect behaviors such as hand raising, head bowing, and listening. Zheng et al. [5] used an improved Faster R-CNN model to automatically detect student behaviors in a real classroom. Zhang et al. [6] improved SlowFast (a 3D CNN) to detect seven common classroom behaviors. Although the CNN and its variants have a high detection accuracy, their real-time performance could be poor due to the two-stage detection framework. Lin et al. [7] proposed a student behavior recognition system based on skeleton pose estimation, utilizing the OpenPose framework to collect skeleton data. Su et al. [8] proposed an improved OpenPose model that can detect six types of student behaviors, such as looking up, looking down, and raising hands. Although skeletal essential point detection techniques improve model accuracy and speed, they require higher computational resources, increasing overall costs. Dong et al. [9] improved the Single Shot MultiBox Detector (SSD) model to detect five behaviors with an average accuracy of 95.4%. Tan et al. [10] used the improved YOLOv3 model to achieve real-time accurate detection of abnormal student behavior in classroom monitoring. Liu et al. [11] proposed the improved YOLOv8n_BT model to detect small target behaviors of occluded and back-row students. The SSD and YOLO models have been widely used for behavioral detection due to their high real-time performance, with SSD detecting with higher accuracy and YOLO detecting with faster speed. Considering the demand for real-time performance in this study, the YOLO model, which is more suitable for this scenario, was chosen.

## 2. Related Work

Since its release in 2016, the YOLO model series has undergone several iterations and improvements and has gradually become the mainstream framework in the field of target detection. YOLOv1 was first proposed by Joseph Redmon’s team in 2016, which innovatively realizes end-to-end learning from the full-image input to the bounding-box output. Although its accuracy is low and it performs poorly, especially in small and dense target detection, its ultra-fast detection speed laid the foundation for the YOLO series. In 2017, YOLOv2 was released by Joseph Redmon’s team. It improves on the shortcomings of YOLOv1 and enhances the detection performance by introducing techniques such as batch normalization, anchor frames, and dimensional clustering. Nevertheless, YOLOv2 does not solve the problem of multi-scale feature fusion and still has limitations in detecting tiny targets. YOLOv3 was introduced by the Redmon and Farhadi team in 2018; it further optimizes the feature extraction network, adds multi-scale detection and residual connectivity, and significantly improves the detection of small targets. However, the model’s application on embedded devices is limited and the accuracy may be degraded in dense target scenarios. In 2020, YOLOv4 was released by Alexey Bochkovskiy, which optimizes the network structure, achieves the best balance between performance and speed, and achieved SOTA performance on the COCO dataset. However, YOLOv4 still has limitations in small target detection. YOLOv5 was released by the Ultralytics team in 2020, who focuses on improving the model’s ease of use and scalability. YOLOv5 offers several lightweight versions that significantly improve detection speed and accuracy and have become widely used in the industry. YOLOv6 was released by Meituan in 2022, who focused on industrial scenarios’ needs and achieved a better speed and accuracy balance. However, its detection accuracy decreases in scenes with significant changes in light and attitude. YOLOv7, released in 2022 by the development team of YOLOv4, focuses on improving detection speed and accuracy but does not perform as well as the other versions in complex scenes and small target detection. YOLOv8, released by Ultralytics in 2023, is an improved version based on YOLOv5. YOLOv8 further optimizes the balance between inference speed and accuracy, making it an efficient and robust framework that is especially suited for real-time detection tasks. Moreover, YOLO11, the latest version of Ultralytics released in 2024, is optimized based on YOLOv8. In the feature fusion network, YOLO11 replaces the C2f module with a more sophisticated C3k2 module and introduces the C2PSA module in the feature extraction network to improve feature extraction. Although YOLO11 enhances the feature extraction capability through these improvements, this complex structure may lead to overfitting in classroom behavior detection, affecting the model’s performance in real applications. On the contrary, the classical neck network used by YOLOv8 is more efficient in dealing with subtle changes in classroom behavior datasets. As a result, in classroom behavior detection tasks, YOLOv8 shows superior detection accuracy and speed. It is especially capable of quickly and accurately identifying student behaviors in complex scenarios with dense student populations and diverse behaviors.

**Figure 1:**
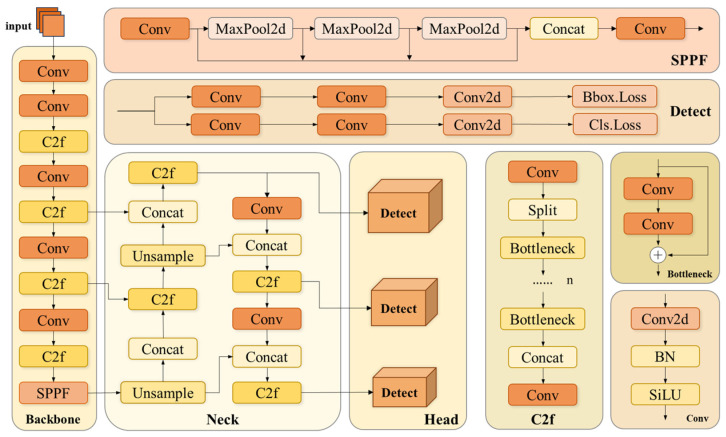
Network structure diagram of YOLOv8.

The structure of the YOLOv8 model is shown in Figure 1, which mainly consists of three parts: the backbone network (Backbone), the neck network (Neck), and the detection head (Head). The backbone network processes the input image through multiple Conv and faster implementation of CSP Bottleneck with two convolutions (C2f) modules to extract feature maps at different scales. The neck network is then responsible for fusing the features extracted by the backbone network and adopting the PANet structure (Path Aggregation Network [12]). PANet facilitates the information flow between the upper- and lower-level features through top-down and bottom-up cross-layer connectivity, thus realizing the effective fusion of features. Finally, the three output branches of PANet are fed into the detection head, which adopts a decoupled head structure to separate the regression branch from the prediction branch and obtains the category and location information of the target object from the feature maps of different scales to complete the task of target classification and localization.

**Figure 2:**
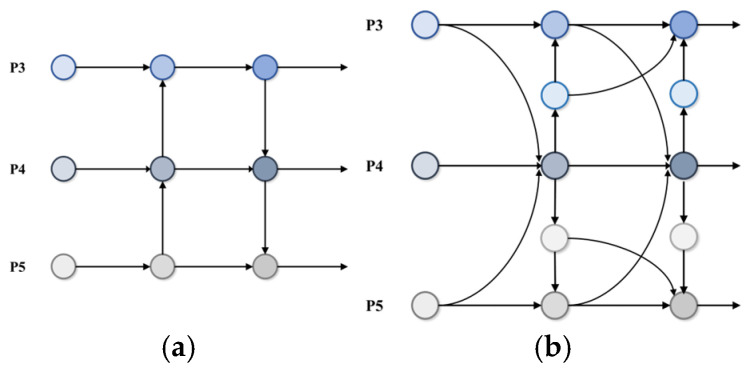
Comparison of feature fusion network structure before and after optimization: (**a**) (YOLOv8); (**b**) (YOLO-AMM).

However, in practical applications, YOLOv8 still faces some challenges in classroom behavior detection tasks, mainly in the following aspects:

1. In student-intensive classroom scenarios, uneven lighting, differences in student morphology, and occlusion problems make it difficult for the model to capture key detailed features. In particular, the model cannot fully utilize the detailed information in the feature maps when there is insufficient multi-scale and multi-level feature fusion. This deficiency further weakens the model’s ability to perceive detailed features and increases the difficulty of behavior recognition.
2. Student behaviors in classroom environments are highly diverse, which requires models to have more substantial feature representation capabilities. However, the feature fusion network structure of the existing model is relatively homogeneous (e.g., Figure 2a), which makes it difficult to fully mine and fuse multi-dimensional feature information from different levels and scales. This weakens the correlation between features, limiting the model’s performance in complex behavior recognition tasks.
3. Differences in the distance between students and the camera lead to variations in scale features, with students at close distances being more precise. In contrast, those at far distances may be blurred or shrunken. The existing model’s detection head needs to improve in dealing with multi-scale targets. It can effectively deal with complex scale variations, affecting the model’s accuracy in capturing features and recognizing students’ behaviors at different distances.

This paper proposes an improved YOLO-AMM model based on YOLOv8 to overcome the above problems. The model is optimized based on YOLOv8 to improve recognition accuracy and speed and to better adapt to the complex scenarios of classroom behavior detection. The main contributions of this study are as follows:

1. In response to the problem of insufficient feature fusion leading to the degradation of detailed feature-capturing ability, we construct the AEFF module. This module enhances the fusion of semantic information between different features and improves the attention to detail features. This optimization effectively enhances the model’s ability to perceive multi-dimensional features, thus improving the model’s recognition of classroom behaviors.
2. To enhance the model’s feature representation ability, we designed the MFFN structure (shown in Figure 2b). This module is synergistically designed to mine and fuse multi-dimensional features from different levels and scales through a multi-scale feature aggregation module and a contextual information diffusion mechanism. This optimization enhances the correlation between different features and makes the model perform more accurately in complex classroom behavior recognition tasks.
  Compared with other feature fusion network structures, MFFN has significant advantages when dealing with features on different scales and levels. Traditional feature fusion methods usually rely on simple feature connectivity or weighted averaging, which often fails to fully utilize the information of the features at each level in variable classroom scenarios. For example, FPN [13] fuses different levels of features through top-down and horizontal connections, but its feature fusion mechanism is relatively simple and lacks deep contextual information mining. PANet adds bottom-up path enhancement to FPN, which improves the feature aggregation capability but still relies on traditional convolutional operations and fails to effectively dig deeply into multi-scale and contextual information. EfficientDet [14], on the other hand, adopts a weighted bidirectional feature pyramid (BiFPN), which excels in efficiency and scalability but may not be as fine as MFFN in capturing detailed features in complex scenes. AFPN [15] reduces information loss through progressive fusion, and although similar to MFFN, MFFN further enhances the fusion of multi-scale features and the diffusion of contextual information through the MSFA and CIDM modules. AFF-FPN [16] introduces the attention mechanism to enhance the feature fusion effect. However, MFFN is more accurate in capturing the details of complex behaviors through a more comprehensive feature aggregation and diffusion mechanism. ABFPN [17], on the other hand, focuses on detecting small targets, whereas MFFN can better deal with the diversity and detail information in complex behaviors.
  MFFN exhibits more vigorous feature representation and detection accuracy in classroom behavior detection tasks through its unique multi-scale feature aggregation and contextual information diffusion mechanism. It is more suitable for handling complex student behavior detection tasks than other network structures.
3. To address the shortcomings of the YOLOv8 detection head in multi-scale target processing, we propose the MSPF-Head module. This module enhances the detection head’s ability to adapt to different-scale targets by optimizing the extraction, fusion, and interaction of multi-scale features. This optimization significantly improves the detection accuracy and speed of the model in complex scenes.

## 3. Improved Methods

**Figure 3:**
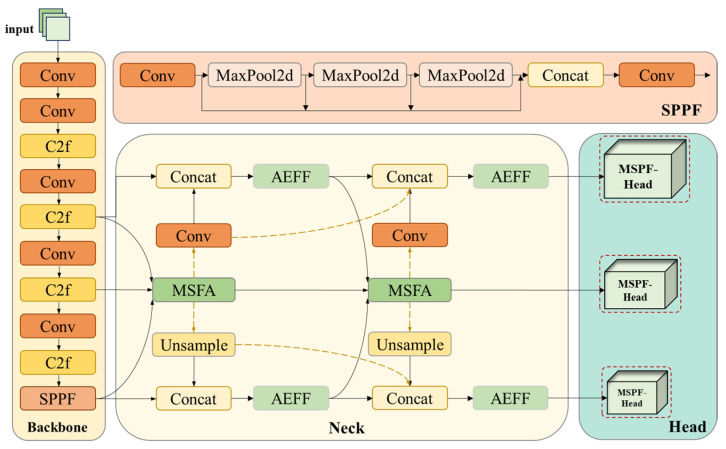
Network structure diagram of YOLO-AMM.

Based on the YOLOv8 framework, we propose a classroom behavior detection model named YOLO-AMM based on multi-dimensional feature optimization. The model achieves real-time detection while maintaining a high detection accuracy. Figure 3 shows the overall architecture of the YOLO-AMM model.

The YOLO-AMM model is inherited from YOLOv8 and consists of three parts: the backbone network (Backbone), the neck network (Neck), and the head network (Head). The Backbone still processes the input image through multiple Conv and C2f modules to extract feature maps at different scales. The Neck, on the other hand, introduces a new feature fusion module, AEFF. This module enhances the fusion capability of semantic information between features by self-adapting the fusion method of different layers of features through LDConv. Meanwhile, during the fusion process, AEFF further enhances the ability to capture detailed features through ELA, thus effectively improving the model’s detection accuracy and detection speed. In addition, YOLO-AMM proposes a new network structure, MFFN, to replace the network structure of the original neck. The MFFN structure optimizes the feature fusion process by combining the multi-scale feature aggregation (MSFA) module and the contextual information diffusion mechanism (CIDM). Among them, the MSFA module can aggregate features at different scales to enhance the model’s ability to adapt to multi-scale targets, thus improving its recognition accuracy and speed for targets of various sizes. CIDM, on the other hand, facilitates the flow of contextual information between different scales so that the lower-level features can be supported by the higher-level semantic information, thus strengthening the correlation between multi-scale and cross-level features. CIDM is a way of connecting different layers of feature information, represented by the yellow dotted line in Figure 3. Such a structural design enhances the depth and breadth of feature expression. It significantly improves the network’s recognition ability in classroom behavior detection. The features processed by the neck form three output branches to be sent to the head. YOLO-AMM uses the MSPF-Head to replace the traditional detection head (shown by the red dashed box in Figure 3). The MSPF-Head can significantly improve the head’s adaptability to targets at different scales through the introduction of the multi-scale sensing, feature interaction, and fusion mechanism, which can achieve a higher detection accuracy.

### 3.1. AEFF Module Design

**Figure 4:**
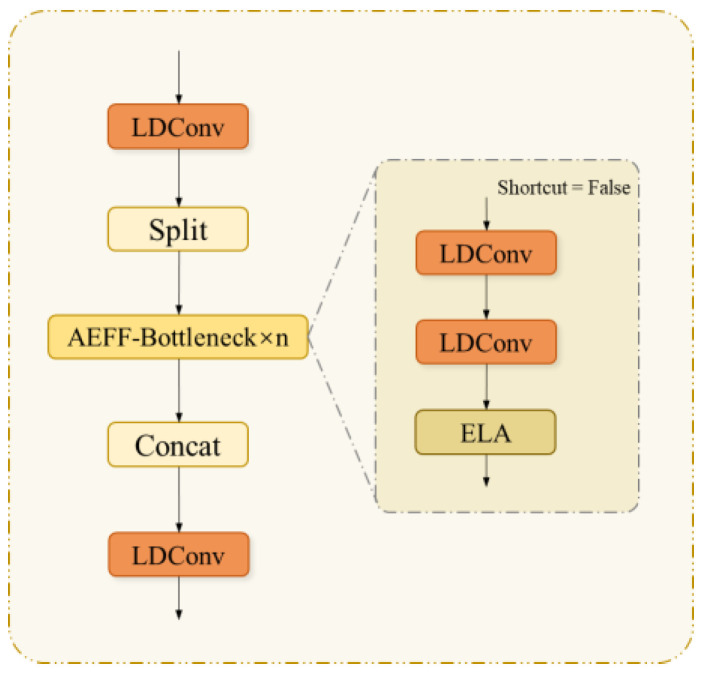
AEFF structure diagram.

This paper proposes a novel feature fusion module, AEFF (structure shown in Figure 4), to replace the C2f module in the original neck network. This module combines Low-parameter Dynamic Convolution (LDConv [18]) and Efficient Local Attention (ELA [19]) mechanisms, significantly enhancing the depth and efficiency of feature fusion and improving the model’s attention to detailed features and ability to fuse semantic information.

#### 3.1.1. Introducing LDConv

In classroom behavior detection, the target features in the surveillance screen may change significantly due to the lighting changes, students’ morphological differences, and the degree of occlusion, which poses a more significant challenge in the feature fusion stage. During the fusion process, standard convolution (SC) cannot adapt to the dynamic changes in target features or adaptively adjust the sampling position. This limitation affects the accuracy of target localization and may increase the risk of false detections. In addition, SC’s deficient sensory field adjustment capability may not be able to effectively capture the fine-grained features of the target, thus triggering the loss of detailed information and further affecting the detection performance of the model.

**Figure 5:**
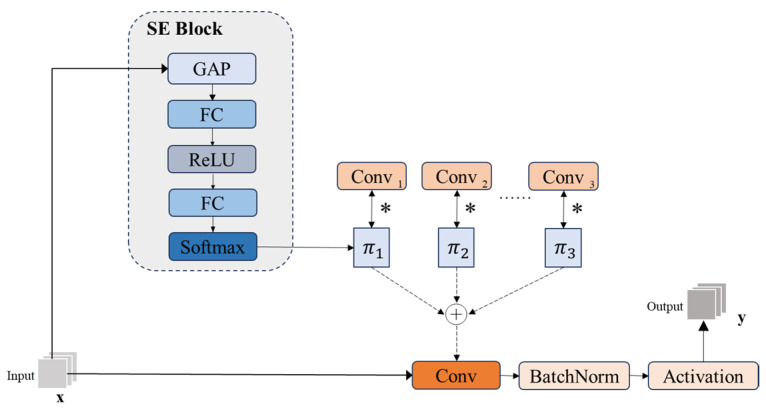
LDConv structure diagram.

In response to standard convolution (SC) limitations, this paper introduces LDConv to adapt to scene-specific challenges. The core of LDConv lies in the generation and application of dynamic convolutional kernels. Unlike traditional convolution that uses a single static convolution kernel, LDConv generates an adaptive dynamic convolution kernel by introducing multiple parallel static convolution kernels, which are dynamically aggregated according to the changes in the input features. The specific implementation process is shown in Figure 5, where LDConv generates the attention weights of the static convolutional kernel through the SE module (Squeeze-and-Excitation Block [20]). Firstly, the SE module uses global average pooling to spatially compress the input features. Two fully connected layers and a nonlinear activation layer are used for feature dimensionality reduction. Finally, the normalized attention weights are obtained through Softmax, $\pi_{k}$. Then, these weights are weighted and summed with the corresponding static convolution kernel to generate the adaptive dynamic convolution kernel, as shown in the following equation:

(1) $$z_{1}=W_{1}\cdot GAP(x),x\in {\mathbb{R}}^{C}$$

(2) $$z_{2}=ReLU(z_{1})=max(0,z_{1})\in [0,+\infty )$$

(3) $$z=W_{2}\cdot z_{2}$$

(4) $$\pi_{k}=Softmax{\left(z\right)}_{k}=\frac{e^{z_{k}}}{\sum_{i=1}^{K}e^{z_{i}}}\in (0,1)$$

(5) $${\tilde{W}}_{dynamic}=\sum_{k=1}^{K}\pi_{k}\cdot {\tilde{W}}_{k}$$

(6) $$y=Activation(BN({\tilde{W}}_{dynamic}*x))$$

In Equations (1)–(4), *GAP*(*x*) denotes the global average pooling over the input feature *x*; $W_{1}$ and $W_{2}$ are the fully connected layer parameters; and $\pi_{k}$ is the attention weight. In Equation (5), ${\tilde{W}}_{k}$ denotes the kth static convolutional kernel; the range of summation is k=1,2,3,⋯,K, where K is the total number of static convolutional kernels and ${\tilde{W}}_{dynamic}$ is the generated dynamic convolutional kernel; * denotes the weighted sum.

Through this nonlinear dynamic aggregation, LDConv significantly improves the flexibility and robustness of feature representation. It not only enhances the ability to perceive key regions in the feature map but also better adapts to the target’s positional changes and regional characteristics, thus realizing more accurate semantic information extraction and fusion and comprehensively improving the detection performance of the model.

#### 3.1.2. Incorporating ELA

In classroom behavior detection, camera resolution limitations pose a challenge to the accuracy of student behavior recognition, especially when subtle behavioral changes in complex backgrounds are more difficult to identify. In addition, detailed information is easily weakened in the feature fusion process. For this reason, this module introduces the ELA mechanism to enhance the model’s ability to pay attention to students’ minute movements and detail changes.

**Figure 6:**
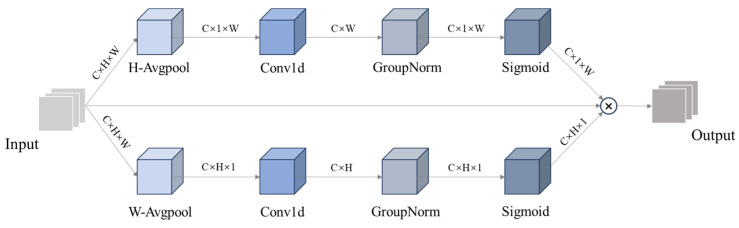
ELA structure diagram.

As shown in Figure 6, ELA employs strip pooling [21] in the spatial dimension to extract feature vectors in the horizontal and vertical directions. By maintaining a narrow kernel shape, ELA can capture long-distance dependencies and prevent extraneous regions from influencing the label predictions. This results in rich target location features in the respective directions. ELA processes the feature vectors in each of the above directions independently to generate the corresponding attention predictions and then subsequently combines these predictions through a multiplication operation to ensure the accurate location information of the target region. Specifically, in the second step, a one-dimensional (1D) convolution is applied to interact locally with the two feature vectors separately, and the coverage of the local interaction is controlled by adjusting the convolution kernel size. The obtained feature vectors are subsequently processed by group normalization (GN [22]) and nonlinear activation functions to generate positional attention predictions in both directions. The final positional attention is obtained by multiplying the positional attention in both directions.

ELA avoids the loss of detailed information by accurately localizing key detail features while effectively suppressing the interference from complex backgrounds. This mechanism improves the model’s attention to detail features, thus enhancing the accuracy of behavior recognition and providing more robust support for behavior detection in complex scenes.

### 3.2. MFFN Structure Design

In classroom behavior detection, student behavior is diverse, covering many different actions. This diversity requires the model to capture subtle and complex features with a more substantial feature expression ability to identify different student behaviors accurately. However, the feature fusion network structure of YOLOv8 is relatively homogeneous. Although its feature fusion mechanism combines shallow and deep information, the network tends to weaken insignificant features in the feature extraction process. This can lead to some detailed information not being fully expressed, thus affecting the accurate recognition of complex behaviors.

**Figure 7:**
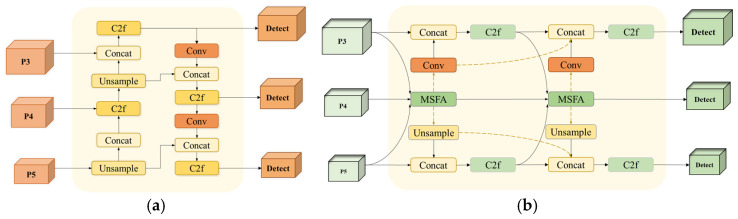
Comparison of feature fusion network structures: (**a**) Original feature fusion network structure; (**b**) MFFN structure.

For this reason, this paper proposes MFFN to optimize the structure of the feature fusion network. The core of the module lies in the Multi-Scale Feature Aggregation Module (MSFA) module and Contextual Information Diffusion Mechanism (CIDM). By working together to optimize the network structure, the module can deeply mine and fuse multi-dimensional feature information from different levels and scales, thus enhancing the model’s feature representation capability in complex classroom behavior detection. The structure of MFFN is shown in Figure 7.

**Figure 8:**
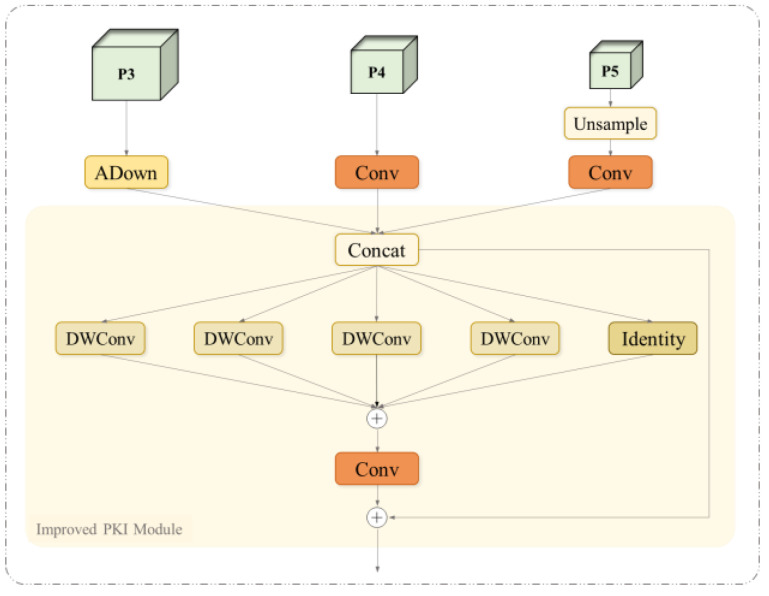
Internal structure of MSFA.

Specifically, the MSFA first receives feature maps from three scales and undergoes up-sampling, adaptive down-sampling (Adown [23]), and 1 × 1 convolution operations for initial channel number adjustment and feature extraction. The ADown module can retain as many target features as possible without compromising the detection accuracy. Subsequently, the extracted features are fed into the improved Poly Kernel Inception (PKI) module [24]. This module extracts local detail features and global context information through a parallel Depthwise Convolution (DWConv) set with effective multi-scale feature fusion. The specific structure of the MSFA module is shown in Figure 8. CIDM, on the other hand, enables the feature maps at each scale to fuse richer semantic information by facilitating the flow of contextual information across detection scales. CIDM is a way of connecting between different layers of feature information, represented by the yellow dotted line in Figure 7b. The mechanism back-propagates high-level semantic information to low-level feature maps using level-by-level feature interactions, thus enabling the network to obtain multi-dimensional features from different levels and scales.

The design of MFFN significantly improves the feature representation capability of the model by focusing on key features and facilitating the flow of information across scales and hierarchies, further enhancing the network’s ability to recognize complex student behaviors. The experimental results showed that adding the MFFN module improved the mean Average Precision (mAP) of model detection by 1.8% compared to the original YOLOv8, which validates the module’s effectiveness.

### 3.3. MSPF-Head Module Design

**Figure 9:**
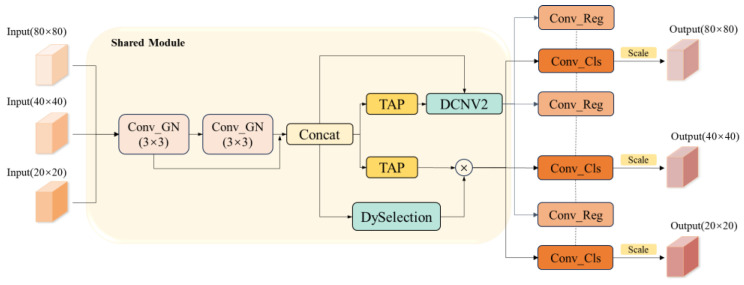
MSPF-Head structure diagram.

To address the shortcomings of the YOLOv8 detection head in multi-scale target processing, we propose the Multi-Scale Perception and Fusion Head (MSPF-Head). The original detection head adopts a single-scale prediction structure, which ignores the importance of multi-scale features for target detection. Meanwhile, the decoupled head structure of YOLOv8 separates the classification and localization tasks with an insufficient task interaction capability. This will prevent the detection head from fully obtaining multi-scale features, resulting in a degradation of detection performance. To solve the above problems, MSPF-Head was optimized in three aspects: multi-scale sensing, feature interaction, and multi-scale fusion, which effectively improve the detection head’s ability to adapt to targets at different scales, thus increasing detection accuracy and speed. The internal structure of MSPF-Head is shown in Figure 9.

MSPF-Head first introduces the group normalization (GN) layer instead of the traditional batch normalization (BN) layer to extract and model the multi-scale features of the inputs. The GN layer improves the multi-scale sensing ability of the detection head so that it can capture rich multi-scale information and it mainly performs better in detecting feature changes of small targets at a long range and large targets at close range. Subsequently, the MSPF-Head splices the extracted multi-scale features in the channel dimension to generate the multi-scale interactive features [25] required for the classification and localization tasks and extracts the multi-scale classification features and localization features through the Task-Aligned Predictor (TAP).

In the localization branch, Deformable ConvNets version 2 (DCNv2 [26]) is introduced for simultaneous adaptive tuning of localization features and fusion of interaction and localization features. DCNv2 adaptively adjusts the convolution kernel by learning dynamic offsets and weight masks to enhance the model’s adaptive ability to different scales of targets and attitude changes. Based on multi-scale fusion, the interaction features provide supplementary information for the localization features, thus further optimizing the expression of localization features. Eventually, the detection head can capture more adequate localization information, effectively improving the accuracy and robustness of target localization.

The classification branch borrows the dynamic idea of DyHead [27], and dynamic selection (DySelection) is set for multi-scale interaction features. Specifically, the dynamic selection module dynamically adjusts the channel weights of the input feature maps through global average pooling, feature mapping, nonlinear activation, and attention weight generation to enhance the detection head’s ability to perceive targets at different scales. Subsequently, the classification features are fused with the selected interaction features to form more comprehensive multi-scale classification features.

To further enhance the detection head’s ability to adapt to multi-scale targets, MSPF-Head incorporates a Scale layer in the output stage. The Scale layer scales the specific outputs of each detection head to optimize the multi-scale feature representation, enabling the model to exhibit superior detection accuracy when dealing with both large and small targets.

In summary, MSPF-Head achieves efficient classification and accurate localization of targets at different scales through multi-scale perception optimization, feature interaction enhancement, and multi-scale fusion improvement. Meanwhile, MSPF-Head also significantly improves the model’s detection accuracy and real-time performance in complex scenes, providing an efficient solution for student behavior detection in complex scenes.

## 4. Experiments

### 4.1. Experimental Details

#### 4.1.1. Dataset

The experiment utilized the publicly available POCO dataset, which was designed to be used in the evaluation of student classroom behavior detection methods. The dataset comprises video data of students’ classroom activities captured by classroom cameras. After data augmentation, the POCO dataset includes 5709 images, categorizing students’ classroom states into three types: active, passive, and neutral, as shown in Table 1. It encompasses a variety of behaviors, such as listening, reading, writing, chatting, sleeping, and using mobile phones. The three neutral states are defined as follows:

1. The student sits straight, possibly listening to the lecture.
2. The student’s cell phone is placed on the desktop without being touched, and the student may be looking at it.
3. The student’s book is on the desktop without being touched, and the student may not be looking at it.

In this experiment, the dataset was randomly divided into training and test sets at a ratio of 9:1 for model training and testing to evaluate the performance of the proposed framework. All data preprocessing operations (e.g., normalization, feature selection, etc.) were only performed on the training set during data division. In contrast, the statistics of the training set were applied to the test set to ensure that the test set samples do not leak into the training set. In addition, a stratified sampling method was used in the division to ensure that the proportion of samples in each category was the same in both the training and test sets, which avoids the bias that the uneven distribution of categories may cause and thus ensures the fairness of the model evaluation.

#### 4.1.2. Evaluation Metrics

To objectively evaluate the performance of the student behavior detection model, the following evaluation metrics are used in the experiments: Precision (P), Recall (R), Average Precision (AP), mean Average Precision (mAP), and Frames Per Second (FPS). Among these metrics, P, R, AP, and mAP represent detection accuracy, and larger values indicate a higher model detection accuracy. FPS represents the detection speed, and larger values indicate faster detection. These metrics were calculated using Equations (7)–(11).

(7) $$Precision(P)=\frac{TP}{TP+FP}\times 100\%$$

(8) $$Recall(R)=\frac{TP}{TP+FN}\times 100\%$$

(9) $$Average\;Precison(AP)=\int_{0}^{1}P(R)dR\times 100\%$$

(10) $$Mean\;Average\;Precison(mAP)=\frac{1}{N}\sum_{i=1}^{N}AP_{i}$$

(11) $$FPS=\frac{1000}{Preprocess+Inference+Postprocess}$$

In these formulas, TP is the number of correctly predicted positive samples, TN is the number of correctly predicted negative samples, FP is the number of negative samples categorized as positive samples, and FN is the number of positive samples categorized as negative samples. P(R) is the precision at a recall rate of R, AP_i_ denotes the Average Precision for the i-th category, and N is the number of behavioral categories. Preprocess is the preprocessing time of the model, which is the time consumed by the image scaling, padding, and channel transformation. Inference is the inference time of the model, which is the time from the preprocessed image input to the model output result. Postprocess is the postprocessing time of the model, which is the time spent on the linear transformation of the model output result.

#### 4.1.3. Experimental Setup

The hardware platform and environment parameters used in the training phase of the experiment are shown in Table 2. The number of training iterations was set to 300 to ensure that the model fully learns the training data, while the Patience parameter was combined to prevent overfitting. The batch size was set to 16 to optimize computational efficiency and memory usage, while the input image size was adjusted to 640 to provide sufficient detail for enhanced detection accuracy. The Workers parameter was set to 8 to speed up the data preprocessing process and to improve the data loading efficiency. The optimizer selected Stochastic Gradient Descent (SGD), and the Momentum parameter was adjusted to 0.937 to accelerate convergence and enhance training stability. The learning rate was set to 0.01 and the learning rate decay factor was set to 0.01 to gradually reduce the learning rate and help the model converge stably. The regularization coefficient was set to 0.0005 to avoid overfitting. In addition, the threshold for turning off Mosaic enhancement was set to 10 to make the model more adaptable under different training conditions.

### 4.2. Evaluation of the YOLO-AMM Model Based on the POCO Dataset

**Figure 10:**
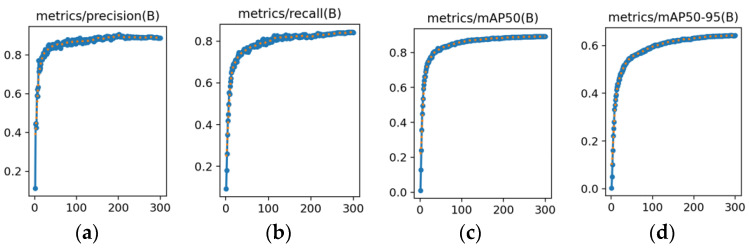
Model training result curves: (**a**) Precision curve; (**b**) Recall curve; (**c**) mAP50 curve; and (**d**) mAP50-95 curve.

When training the network model for student behavior detection, the model was optimized using a Stochastic Gradient Descent (SGD) optimizer. The mosaic data enhancement technique was used in the last ten training stages. This adjustment aims to improve the model’s robust performance in detecting student behavior. The training results are shown in Figure 10.

In Figure 10, each subplot represents the change in different key evaluation metrics during the training process of the YOLO-AMM model. The horizontal axis represents the number of training rounds, and the vertical axis represents the values of the corresponding metric in each training round.

- (a) Precision curve: Accuracy measures the percentage of correctly identified targets in the model detection results and is defined as shown in Equation (7).
- (b) Recall curve: Recall reflects the model’s ability to cover positive samples that actually exist, which is defined as shown in Equation (8).
- (c) mAP50 curve: mAP50 is the average detection accuracy of the model when the IoU (intersection and merger ratio) threshold is 0.5, which comprehensively evaluates the detection performance of all categories. Its calculation formula is shown in Equations (12) and (13).
  (12) $$AP_{i}^{50}={\int_{0}^{1}P_{i}(R)dR\times 100\%}_{IoU=0.5}$$
  (13) $$mAP_{50}=\frac{1}{N}\sum_{i=1}^{N}A{P_{i}}^{50}$$
- (d) mAP50-95 curve: mAP50-95 evaluates the comprehensive performance of the model under stringent localization requirements by calculating the average accuracy for IoU thresholds ranging from 0.5 to 0.95 (in steps of 0.05). Its calculation formula is shown in Equations (14)–(16).
  (14) $$AP_{i}^{\theta_{k}}={\int_{0}^{1}P_{i}(R)dR\times 100\%}_{IoU=\theta_{k}}$$
  (15) $$A{P_{i}}^{50-95}=\frac{1}{10}\sum_{k=0}^{9}A{P_{i}}^{\theta_{k}}$$
  (16) $$mAP_{50-95}=\frac{1}{N}\sum_{i=1}^{N}A{P_{i}}^{50-95}$$

As the number of training rounds increased, all the curves gradually increased and stabilized, indicating a steady improvement and eventual convergence of the model’s performance. These results show that the YOLO-AMM model effectively improved the detection accuracy, confirming its stability and effectiveness in training and validation.

### 4.3. Ablation Experiment

This section analyzes the contributions of AEFF, MFFN and MSPF-Head to the model performance through ablation experiments. Each module was designed based on the theoretical analysis of common problems in classroom behavior detection tasks, aiming to improve the model’s feature fusion efficiency, enhance the model’s feature representation, and optimize the model’s ability to handle multi-scale targets. In the following experiments, we analyzed the performance changes after adding each module individually, and discuss its impact using a theoretical analysis. In Table 3, “√” indicates the optimization module that was added to the YOLOv8n network model.

In this experiment, we replaced the original C2f module in the feature fusion stage of the YOLOv8n model with the AEFF module. The AEFF module enhances the semantic information fusion between features by adaptively adjusting the fusion of features at different levels and, in the process, improves the ability to capture detailed features, thus effectively improving the detection accuracy and detection speed of the model. Specifically, AEFF generates a dynamic convolutional kernel through LDConv, adapts to the changes in target features, and dynamically selects the optimal feature fusion strategy. In addition, AEFF further strengthens the attention to detailed features through ELA. The core of ELA lies in extracting horizontal and vertical features separately through strip pooling, processing them independently, generating positional attention predictions, and ultimately combining the attention in both directions to capture the target position accurately. By weighting the local regions, ELA effectively highlights the detailed features and suppresses the background noise to improve the accuracy of AEFF in capturing the detailed features. Without AEFF, the feature fusion process of the model is relatively single, and the standard convolution is challenging to adapt to the dynamic changes in the target features, which can easily lead to the fusion of redundant or irrelevant features, thus increasing the amount of computation and affecting the detection accuracy and speed. The experimental results showed that after adding the AEFF module, the model improved in several indicators. Specifically, compared with the initial YOLOv8n model, the model with AEFF improved by 0.8%, 1.0%, 1.0%, 1.0%, 1.0%, and 1.0% in Precision (P), Recall (R), mAP50, and mAP50-95, respectively, and the FPS improved by 19.3% to 186.3 frames per second. This result validates the key role of the AEFF module in improving detection accuracy and speed.

Next, we replaced the original feature fusion network structure in the YOLOv8n model with the MFFN structure, which optimizes the feature fusion process using the multi-scale feature aggregation (MSFA) module and the contextual information diffusion mechanism (CIDM). The MSFA module enhances the model’s adaptability to multi-scale targets by aggregating features at different scales, especially in complex scenarios; it can effectively integrate information from different scales and enhance the model’s recognition accuracy and speed for targets of various sizes. CIDM, on the other hand, facilitates the flow of contextual information between different scales so that the low-level features can be supported by the high-level semantic information, which strengthens the correlation between multi-scale and cross-level features. This design enhances the depth and breadth of feature representation and significantly improves the network’s recognition ability in classroom behavior detection. By introducing MFFN to optimize the network structure of the feature fusion layer, the model showed significant improvement in several performance metrics. Compared with the original YOLOv8 model, adding the MFFN structure improved Recall (R), mAP50, mAP50-95, and FPS by 1.6%, 0.9%, 1.8%, and 5.5%, respectively. These results show that MFFN can significantly improve the model’s detection accuracy and speed, verifying its effectiveness in feature fusion and information flow optimization.

Next, we replaced the original detection head in the YOLOv8n model with the MSPF-Head. MSPF-Head significantly improves the model’s ability to adapt to targets at different scales by introducing multi-scale sensing, feature interaction, and fusion mechanisms, which improve detection accuracy. Specifically, MSPF-Head has three main structural optimizations: first, group normalization (GN) is used instead of the traditional batch normalization (BN), which improves the multi-scale feature-sensing ability and it mainly performs better in the detection of small targets at a long distance and large targets at a close distance; second, dynamic convolution (DCNv2) is introduced into the localization branch, which is used for the adaptive tuning of localization features, which enhances the model’s adaptability to attitude changes and multi-scale targets; finally, the classification branch further optimizes the fusion of multi-scale interaction features through the dynamic selection module (DySelection), which improves the classification accuracy. Although MSPF-Head led to a slight decrease in FPS in some cases, the detection speed remained above 100 FPS, which is sufficient for real-time detection. Meanwhile, accuracy metrics such as Precision (P), Recall (R), mAP50, and mAP50-95 improved by 1.3%, 1.5%, 1.9%, and 3.0%, respectively. This indicates that MSPF-Head maintains competitiveness in real-time detection capability and significantly improves the model’s detection accuracy in complex scenes by optimizing multi-scale feature sensing and fusion. These results validate the effectiveness of MSPF-Head in enhancing the performance of the detection head, especially in providing more comprehensive feature representation and more accurate target localization when dealing with multi-scale targets.

**Figure 11:**
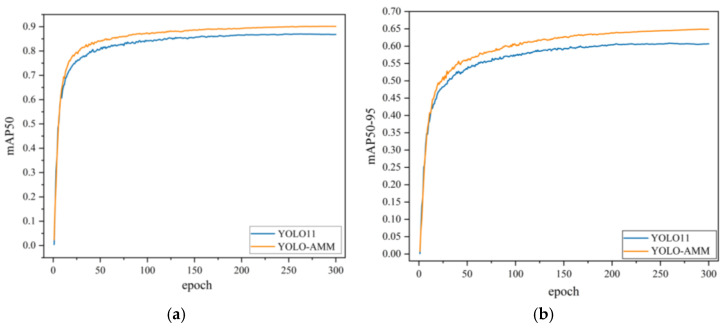
Comparison of mAP values before and after improvement: (**a**) Comparison of mAP50 curves; (**b**) Comparison of mAP50-95 curves.

By fusing these three improved modules, the YOLO-AMM model demonstrated significant advantages over YOLOv8n. Figure 11 shows the accuracy comparison between the original model and the final improved model more intuitively. From the results of the ablation experiments, the three improved modules effectively improve the detection accuracy and detection speed of the whole model, which can now realize high-precision and low-latency real-time detection in complex classroom scenarios.

### 4.4. Comparison Experiment

To further validate the detection accuracy and speed of the models proposed in this paper, we compared ten representative network models: the Faster R-CNN, SSD, YOLOv5n, YOLOv6s, YOLOv7-tiny, YOLOv8n, YOLOv11n, SBD-Net, VWM-YOLOv8, and SimAM-YOLOv8 models. We used the same dataset to train these models. The experimental results are shown in Table 4, using Precision (P), Recall (R), and Mean Accuracy (mAP) as the evaluation metrics for model detection accuracy and the parameter FPS as the evaluation metric for model detection speed.

As can be seen from Table 4, comparing the current popular target detection algorithms, YOLO-AMM was more advantageous based on all the indexes. Compared with Faster R-CNN, SSD, YOLOv5n, YOLOv6s, YOLOv7-tiny, YOLOv8n, YOLOv11n, SBD-Net, VWM-YOLOv8, and SimAM-YOLOv8, the Precision (P) of YOLO-AMM improved by 15.9%, 20.0%, 3.7%, 5.9%, 11.7%, 1.6%, 1.5%, 0.6%, and 1.4%, respectively. The Recall (R) of YOLO-AMM improved by 13.9%, 11.1%, 13.3%, 6.1%, 10.8%, 3.8%, 1.6%, 1.6%, and 3.1%, respectively. The mAP50 of YOLO-AMM improved by 14.8%, 22.2%, 10.3%, 3.5%, 5.6%, 3.1%, 1.6%, 1.5%, and 2.6%, respectively. The mAP50-95 of YOLO-AMM improved by 22.2%, 26.5%, 13.6%, 7.2%, 10.9%, 3.5%, 1.2%, 1.3%, and 3.0%, respectively. The FPS of YOLO-AMM increased by 156.0 fps, 110.9 fps, 30.5 fps, 24.6 fps, 9.3 fps, 12.9 fps, 21.2 fps, 38.7 fps, 90.2 fps, and 40.1 fps, respectively.

The comparison experiments showed that the YOLO-AMM model is more effective in detecting student behavior in classroom scenarios and has an advantage in detection speed. The improved model considers accuracy and real-time design and is highly practical.

### 4.5. Practical Detection Effect

**Figure 12:**
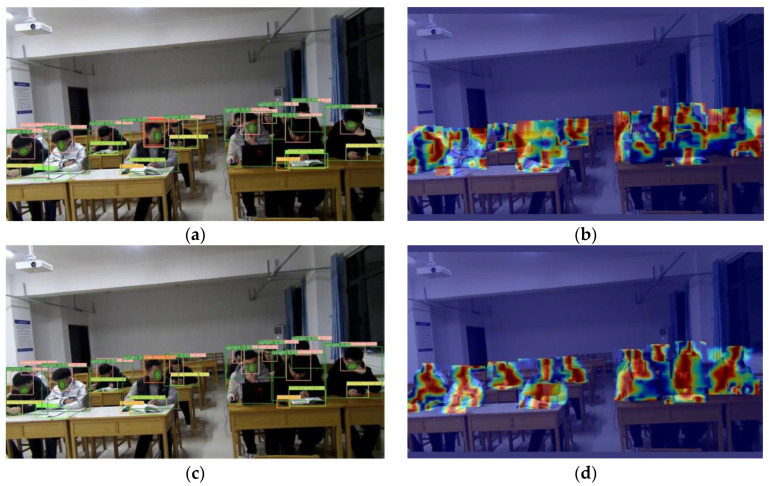
Comparison of sparse behavior detection based on number of students: (**a**) student behaviors detected by YOLOv8 in a dense scene; (**b**) heat map generated by YOLOv8, highlighting the detected areas; (**c**) detection results of YOLO-AMM in the same classroom environment; (**d**) heat map generated by YOLO-AMM. The heat map colors range from blue (weak features) to red (strong features), reflecting the intensity of the features detected by the model.

**Figure 13:**
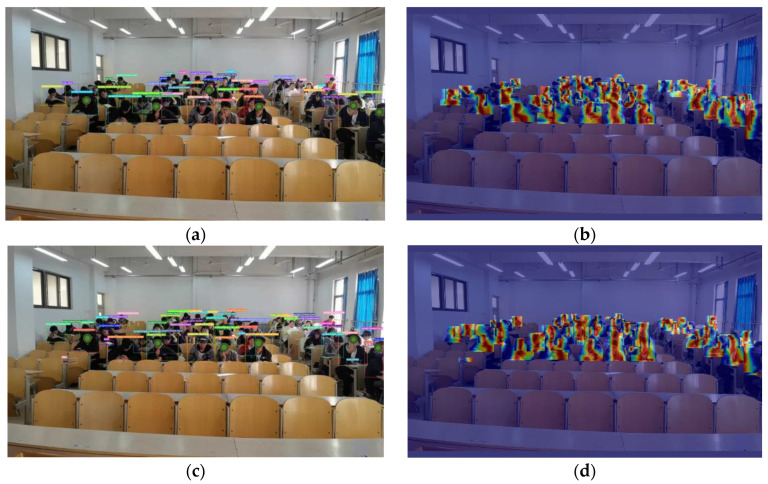
Comparison of intensive behavior detection based on number of students: (**a**) student behaviors detected by YOLOv8 in a dense scene; (**b**) heat map generated by YOLOv8, highlighting the detected areas; (**c**) detection results of YOLO-AMM in the same classroom environment; (**d**) heat map generated by YOLO-AMM. The heat map colors range from blue (weak features) to red (strong features), reflecting the intensity of the features detected by the model.

This study selected and analyzed several image samples from real classroom scenarios to verify the detection performance of YOLO-AMM in real classroom environments. Figure 12 and Figure 13 demonstrate the improvement with YOLO-AMM compared to the traditional YOLOv8n model. In particular, (a) and (c) are detection block diagrams showing the detected student behaviors and detection accuracy, while (b) and (d) are heat maps showing the regions that the model focused on during the detection process.

In a relatively simple classroom scenario, the YOLOv8n model suffered from a high false detection rate and insufficient detection accuracy in the target detection task, especially when dealing with targets with ambiguous behavioral boundaries. In contrast, YOLO-AMM had a significantly improved ability to capture detailed key features by efficiently fusing multi-dimensional features. The improved model could focus more attention on student behavioral regions, effectively reducing the frequency of false detections while significantly improving the overall detection accuracy.

In more complex classroom scenarios (e.g., long-distance targets, occlusion, or complex backgrounds), the YOLOv8n model showed more significant limitations in recognizing long-distance targets and often failed to detect or locate the target accurately. In contrast, by optimizing the multi-scale feature extraction and fusion mechanism, YOLO-AMM enhanced the model’s ability to adapt to targets at different scales with complex backgrounds and significantly reduced the missed detection rate. In addition, the improved model showed stronger robustness and detection stability when coping with occlusions, background interference, and scale changes.

## 5. Conclusions

This paper proposed a real-time classroom behavior detection algorithm based on multi-dimensional feature optimization, YOLO-AMM. We improved the YOLOv8 model with a series of optimizations to overcome the limitations of the traditional target detection model in complex classroom environments. First, we constructed the AEFF module, which improves the model’s ability to capture detailed information by enhancing the semantic fusion between different features and significantly improves the accuracy of student behavior recognition. Second, we designed the MFFN structure, which establishes a closer correlation between feature information at various levels and scales, thus improving the model’s feature expression ability and behavior recognition performance. Finally, we propose the MSPF-Head module, which enhances the adaptability of the detection head to targets at different scales by optimizing the extraction, fusion, and interaction of multi-scale features and improves the detection accuracy and speed.

The experimental results showed that YOLO-AMM improved the detection accuracy by 22.2%, 26.5%, 13.6%, 7.2%, 10.9%, 3.5%, 1.2%, 1.3%, and 3.0% compared with other mainstream target detection models (Faster R-CNN, SSD, YOLOv5n, YOLOv6s, YOLOv7-tiny, YOLOv8n, YOLOv11n, SBD-Net, VWM-YOLOv8, and SimAM-YOLOv8, respectively). Also, YOLO-AMM improved the detection speed FPS by 156.0 fps, 110.9 fps, 30.5 fps, 24.6 fps, 9.3 fps, 12.9 fps, 21.2 fps, 38.7 fps, 90.2 fps and 40.1 fps, respectively. These improvements demonstrate that YOLO-AMM can achieve more efficient and real-time behavioral detection in complex classroom scenarios.

However, although YOLO-AMM demonstrated significant advantages in the current classroom behavior monitoring task, certain limitations still exist. For example, in some extreme environments, the model may be affected by extreme lighting conditions or different camera angles, so future research will further enhance the adaptability of these aspects, possibly combining multimodal data (e.g., infrared, depth images, etc.) to enhance the robustness of the model. In addition, as the complexity of classroom scenarios continues to increase, cross-disciplinary multi-sensor fusion techniques (e.g., acoustic sensors or behavioral analytics data) will also be key to further improving behavioral recognition accuracy.

In summary, YOLO-AMM provides an effective solution for classroom behavior monitoring and lays a solid technical foundation for applying intelligent visual sensors in education. In the future, with the continuous optimization of algorithms and the expansion of application scenarios, YOLO-AMM is expected to become an essential part of the intelligent education environment, promoting the improvement of education quality and the innovation of teaching modes.

## Figures and Tables

**Table 1:** Description of the student behavior dataset.

Label Category	Name of the Behavior	Student Status
front face	listening	positive
bowed head	bowing the head	negative
side head	chatting	negative
upright	sitting upright	neutral
body desk	leaning over the table	negative
phone	phone	neutral
phone hands	playing cell phones	negative
book	book	neutral
book hands	reading/writing	positive
head arms	sleeping	negative

**Table 2:** Training environment and parameter configurations.

Platform	Parameter Configuration
System	Windows11
CPU	Intel(R) Core(TM) i7-14650HX (Intel, Santa Clara, CA, USA)
GPU	NVIDIA GeForce RTX4090 (NVIDIA, Santa Clara, CA, USA)
Development environment	PyCharm 2022.2.5
Compilation environment	Python 3.8
Deep learning framework	Pytorch 1.11.0

**Table 3:** Comparison of ablation experiment results.

AEFF	MFFN	MSPF-Head	P/%	R/%	mAP50/%	mAP50-95/%	FPS/fps
			88.6	80.7	87.0	60.8	156.2
√			89.4	81.7	88.0	61.8	186.3
	√		88.6	82.3	87.9	62.6	164.8
		√	89.9	82.2	88.9	63.8	151.9
√	√		89.5	82.5	88.9	63.3	174.3
	√	√	89.9	83.8	89.3	64.5	158.2
√		√	90.1	83.5	89.8	64.0	165.5
√	√	√	90.2	84.5	90.1	64.8	169.1

**Table 4:** Performance comparison of different models.

Model	P/%	R/%	mAP50/%	mAP50-95/%	FPS/fps
Faster R-CNN	74.3	70.6	75.3	42.1	13.1
SSD	70.2	73.4	67.9	37.8	58.2
YOLOv5n	86.5	71.2	79.8	50.7	138.6
YOLOv6n	84.3	78.6	85.2	59.3	144.5
YOLOv7-tiny	78.5	73.7	84.5	53.4	159.8
YOLOv8n	88.6	80.7	87.0	60.8	156.2
YOLO11n	87.0	79.9	86.4	60.6	147.9
SBD-Net [28]	88.7	82.9	88.5	63.1	130.4
VWM-YOLOv8 [29]	89.6	82.9	88.6	63.0	78.9
SimAM-YOLOv8 [30]	88.8	81.4	87.5	61.3	129.0
YOLO-AMM	90.2	84.5	90.1	64.3	169.1

## Data Availability

The Classroom Behavior POCO dataset was obtained from https://github.com/jwmianzu/POCO-Dataset (accessed on 31 January 2024).
